# Supplementary material for: The Role of Factor Xa-Independent Pathway and Anticoagulant Therapies in Cancer-Related Stroke
Source: J Clin Med. 2021 Dec 27;11(1):123. doi: 10.3390/jcm11010123 (PMC8745325; doi:10.3390/jcm11010123)
Supplement: Supplementary file 1 [file jcm-11-00123-s001.zip › jcm-1497215-supplementary.pdf]

SUPPLEMENTAL MATERIAL

# Role of Factor Xa-Independent Pathway and Anticoagulant therapies in Cancer-related Stroke

Hyung Jun Kim, M.D., Jong-Won Chung, M.D., Ph.D., Oh Young Bang, M.D., Ph.D., Yun Jeong Lim, R.N., Jaechun Hwang, M.D. Woo-Keun Seo, M.D., Ph.D., Gyeong-Moon Kim, M.D., Ph.D., Hee-Jin Kim, M.D., Ph.D., Myung-Ju Ahn, M.D., Ph.D.

**Table S1.** Laboratory findings associated with recurrence of cancer stroke and treatment-related changes.

| | **Situation 1** | **Situation 2** | ***P*** |
|---|---|---|---|
| Recurrence of cancer stroke | Stable | Recurrence | |
| D-dimer, μg/mL (n=10) | 8.80±7.31 | 28.11±21.22 | *0.002* |
| Factor Xa, % (n=9) | 69.11±9.96 | 62.33±18.32 | *0.164* |
| cfDNA, ng/ml (n=6) | 51.56±9.94 | 65.70±13.49 | *0.022* |
| Cancer stroke | Enoxaparin | | |
| | Pre-treat | Post-treat | |
| D-dimer, μg/mL (n=32) | 16.05±13.28 | 8.58±12.26 | *0.011* |
| Factor Xa, % (n=28) | 71.35±25.15 | 71.92±19.56 | *0.858* |
| cfDNA, ng/ml (n=15) | 53.69±18.86 | 52.98±11.41 | *0.495* |
| Cancer stroke | Edoxaban | | |
| | Pre-treat | Post-treat | |
| D-dimer, μg/mL (n=12) | 16.68±10.48 | 13.08±9.43 | *0.071* |
| Factor Xa, % (n=12) | 77.44±15.49 | 68.94±14.61 | *0.019* |
| cfDNA, ng/ml (n=9) | 56.73±6.54 | 71.38±12.30 | *<0.001* |
| AF stroke | Factor Xa inhibitor | | |
| | Pre-treat | Post-treat | |
| D-dimer, μg/mL (n=16) | 1.53±1.76 | 0.37±0.16 | *0.009* |
| Factor Xa, % (n=35) | 81.92±15.10 | 75.71±14.21 | *0.002* |
| cfDNA, ng/ml (n=15) | 41.97±7.75 | 38.05±4.04 | *0.096* |

**Figure S1**. An example case showing D-dimer, factor Xa activity, and cell free DNA levels changes between the occurrence and recurrence of cancer stroke.

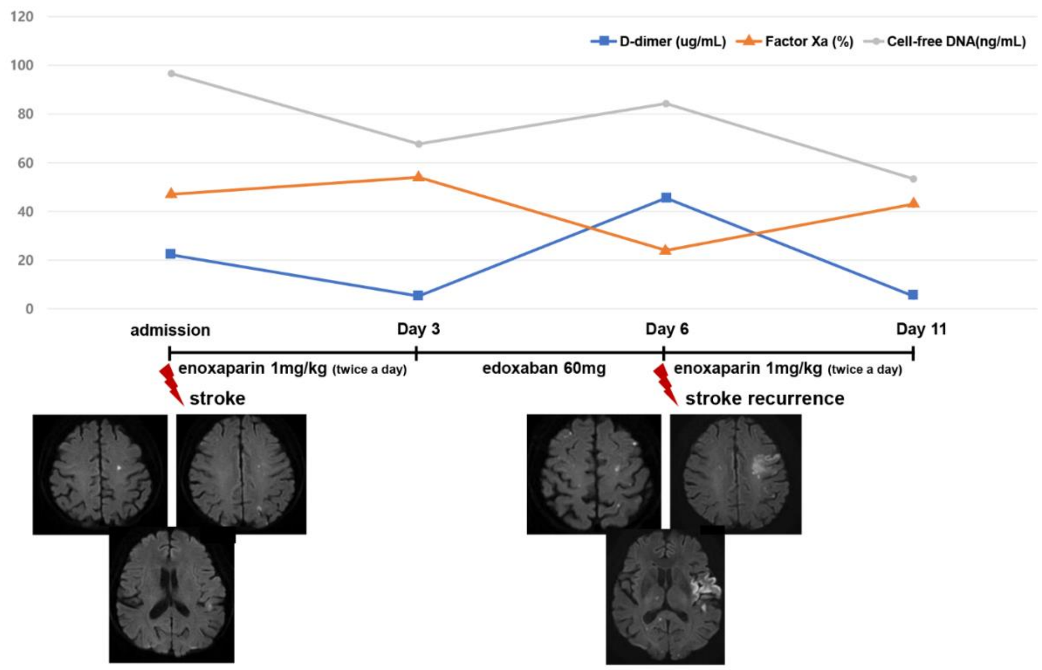


A 65-year-old female patient with pancreatic head cancer and liver metastasis was referred to a neurologist due to sudden disorientation while hospitalized for chemotherapy. After 3 days of receiving 1 mg/kg enoxaparin (twice a day), D-dimer level decreased and the disorientation improved, so she was transferred to edoxaban. After 3 days of receiving edoxaban, worsening of disorientation and right-hand weakness occurred, and stroke recurrence was confirmed by the diffusion-weighted image.
